# Gene Expression Profiling in Abdominal Aortic Aneurysms

**DOI:** 10.3390/jcm11123260

**Published:** 2022-06-07

**Authors:** Amelie L. Behrens, Susanne Dihlmann, Caspar Grond-Ginsbach, Andreas S. Peters, Bernhard Dorweiler, Dittmar Böckler, Philipp Erhart

**Affiliations:** 1 Department of Vascular and Endovascular Surgery, University Hospital of Heidelberg, 69120 Heidelberg, Germany; amelie.behrens@uk-koeln.de (A.L.B.); susanne.dihlmann@med.uni-heidelberg.de (S.D.); caspar.grond-ginsbach@med.uni-heidelberg.de (C.G.-G.); andreas.peters1@med.uni-heidelberg.de (A.S.P.); dittmar.boeckler@med.uni-heidelberg.de (D.B.); 2 Department of Vascular and Endovascular Surgery, University Hospital Cologne, 50937 Köln, Germany; bernhard.dorweiler@uk-koeln.de

**Keywords:** abdominal aortic aneurysm, gene expression, mechanotransduction, rupture risk

## Abstract

Gene expression profiling of abdominal aortic aneurysms (AAA) indicates that chronic inflammatory responses, active matrix metalloproteinases, and degradation of the extracellular matrix components are involved in disease development and progression. This study investigates intra- and interpersonal RNA genome-wide expression profiling differences (Illumina HumanHT-12, BeadCHIP expression) of 24 AAA biopsies from 12 patients using a single gene and pathway (GeneOntology, GO enrichment) analysis. Biopsies were collected during open surgical AAA repair and according to prior finite element analysis (FEA) from regions with the highest and lowest wall stress. Single gene analysis revealed a strong heterogeneity of RNA expression parameters within the same and different AAA biopsies. The pathway analysis of all samples showed significant enrichment of genes from three different signaling pathways (integrin signaling pathway: fold change FC 1.63, *p* = 0.001; cholecystokinin receptor pathway: FC 1.60, *p* = 0.011; inflammation mediated by chemokine signaling pathway: FC 1.45, *p* = 0.028). These results indicate heterogeneous gene expression patterns within the AAA vascular wall. Single biopsy investigations do not permit a comprehensive characterization of activated molecular processes in AAA disease.

## 1. Introduction

Abdominal aortic aneurysm (AAA) is a degenerative disease of the abdominal aorta leading to progressive dilatation. AAA is defined as a maximum diameter of the abdominal aorta to more than 150% of the original diameter (>30 mm in humans). It remains a major cause of morbidity and, if left untreated, can ultimately lead to rupture with lethality rates of up to 80% [1]. To date, clinical prediction of AAA rupture risk and treatment decisions have been based mainly on AAA size and growth rate. Although the risk of rupture increases with aortic aneurysm diameter, the natural history of the disease varies markedly between different patients [2], suggesting different pathomechanisms and challenging the view that AAA diameter and growth rate alone are sufficient for rupture prediction [3,4]. Therefore, a detailed understanding of the pathophysiological processes involved in the development and progression of AAAs is essential to enable future assessment of an individual patient’s risk of rupture.

According to current knowledge, the pathophysiology of AAAs mainly involves chronic inflammatory processes, activation of matrix metalloproteinases (MMPs), and degradation of the extracellular matrix (ECM) [5,6,7].

Elastic fibers and mainly type I and III collagen within the tunica media, ensuring integrity and elastic properties of the aortic vessel wall, are frequently degraded in AAA development. MMPs such as MMP2 and MMP9, and the expression of other destabilizing factors were postulated to be associated with mechanical and morphological remodeling of the AAA wall [3,8].

Biomechanical stress on the aortic wall due to the pulsatile blood flow may affect focal gene expression patterns, a response phenomenon that has commonly been referred to as “mechanotransduction” [9]. Our previous pilot study to analyze gene expression in different AAA biopsies in correlation to biomechanical finite element analysis (FEA) revealed increased expression of genes involved in the degradation of ECM components in AAA regions with calculated peak wall stress (PWS). However, gene expression patterns differed significantly between biopsies of the same and different AAAs [10]. In contrast to single gene expression studies, the heterogeneity of AAA pathogenesis might be better captured by whole-genome expression analyses.

This study investigated the genome-wide gene expression patterns and gene pathways of AAA specimens (1) to investigate whether local gene expression is related to biomechanical parameters from FEA, (2) to compare single gene and pathway gene expression analyses of AAA biopsies, and (3) to interpret these data with respect to previous AAA gene expression results.

## 2. Materials and Methods

### 2.1. Finite Element Analysis and Tissue Sampling

The AAA specimens used in the study were provided by the Vascular Biobank Heidelberg (VBBH). Patients with asymptomatic infrarenal abdominal aortic aneurysms were included in this study. Exclusion criteria were hereditary connective tissue diseases and inflammatory aneurysms. Tissue samples were obtained during elective open surgical aortic replacement. Based on preoperative CT angiography (CT-A), FEA was performed using A4clinics™ software (VASCOPS GmbH, Research Edition, Graz, Austria). Thus, for each area in the aneurysm wall, the individual rupture risk was calculated in terms of peak wall stress (PWS) and color-coded in a three-dimensional reconstruction of each aneurysm. During surgery, AAA wall samples were taken in a clockwise orientation from each of the areas with the highest and lowest PWS, as reported elsewhere [11].

The study sample of the current investigation consisted of a total of 12 patients, i.e., 24 whole-genome RNA expression profiling datasets of AAA specimens were analyzed.

All patients had previously given their informed consent for inclusion before providing the biopsies taken to the VBBH for research purposes. The study was approved by the Ethics Committee of the Medical Faculty Heidelberg S-149/2010, S-301/2013 and amendment of 2016). Patient’s characteristics are summarized in Table 1.

### 2.2. RNA Extraction and Gene Expression Profiling

RNA was extracted from freshly frozen biopsies using the RNeasy Fibrous Tissue Mini Kit (Qiagen, Hilden, Germany) according to the manufacturer’s instructions. A two-step quality and quantity control in terms of spectrophotometric analysis using the NanoDrop 2000c (Thermo Fisher Scientific Inc., Waltham, MA, USA) was performed to select the samples that contained at least 500 ng of RNA at a concentration of at least 30 ng/µL. Gene expression profiling was then performed at the DKFZ (Deutsches Krebsforschungszentrum, Heidelberg, Germany) Genomics and Proteomics Core Facility using Ilumina HumanHT-12 v4 Expression BeadChips (Ilumina, San Diego, CA, USA).

### 2.3. Bioinformatic Analysis of Gene Expression Patterns

Differential gene expression was analyzed between lowest and highest wall stress AAA samples and additionally within the samples of the same AAAs.

Statistical analysis of gene expression data was then performed using XLSTAT by addinsoft (version 2019.4.1, Addinsoft, Paris, France). First, logarithmization (log2) of the transcripts previously normalized using SAM (significance analysis of microarrays) was performed. Further filtering steps were taken to exclude nonspecific bound transcripts (background noise) based on the average of the mean expression values for each transcript (>7.04). Correctly bound, actually expressed transcripts had higher gene expression values across samples compared to mismatched transcripts (standard deviation > 0.16). A paired *t*-test was performed to analyze the expression levels of highest and lowest wall stress AAA specimens. Only transcripts with a nominal (not corrected for multiple testing) *p* < 0.05 were included in further analysis.

Individual gene expression profiling was used to identify transcripts that showed a mean expression difference of at least 20% between the high and low wall stress AAA specimens. Mean expression values of both groups were calculated and de-logarithmized to compare fold changes (FC) >1.2 expression upregulation or FC <0.8 expression downregulation.

As two specimens were collected from each patient, intra-individual gene expression differences were also assessed. After exclusion of transcripts with mean expression differences of FC <1.5 or >0.5 (50% respectively), selected transcripts were ranked according to mean expression differences.

In addition, a pathway analysis was performed in terms of a GeneOntology (GO) enrichment analysis (http://geneontology.org, access date on 1 March 2022) [12,13,14]. Enrichment was analyzed using the PANTHER overrepresentation test. *p*-values < 0.005 after Bonferroni correction for multiple testing were considered statistically significant.

## 3. Results

### 3.1. Individual Gene Expression Profiling

Genome-wide gene expression profiling yielded 48,107 SAM-normalized transcripts. After adjustment and exclusion of unspecific expressed transcripts, individual gene analysis was performed with a total of 14,273 transcripts. In the single-gene analysis, 56 transcripts from 48 genes displayed a mean expression value with a mean value of at least 20% higher in biopsies from tissues with the highest wall stress compared to samples with the lowest wall stress than in the lowest walls stress samples. Table 2 illustrates the highest gene expression candidates in AAA regions with the highest wall stress.

Conversely, 8 transcripts from 7 genes displayed mean expression values higher than 20% in biopsies taken from AAA tissue regions with the lowest wall stress (FC < 0.8) (see Table 3).

Considering gene expression differences within the two biopsies of each patient, a total of 361 transcripts from 322 genes showed a mean expression difference >50%. In particular, transcripts of several MMPs showed a mean expression difference >50%, such as MMP-7 (161.5%, M = 71.6), MMP-9 (124.3%, M = 57.3) and MMP-12 (131.3%, M = 78.1%) (see Table 4).

### 3.2. Pathway Analysis

Of the 14,273 transcripts included in the pathway analysis, 9806 could be mapped. Pathway analysis showed significant enrichment of genes from 3 different signaling pathways (see Table 5). Enrichment was found within the integrin signaling pathway (150 of 199 genes; FC 1.63, *p* = 0.001), the Gastrin and cholecystokinin receptor CCKR signaling map (131 of 172 genes; FC 1.60, *p* = 0.011), and the inflammation mediated by the chemokine and cytokine signaling pathway (176 of 255 genes; FC 1.45, *p* = 0.028). In addition, Gene Ontology (GO) enrichment analysis of all the differentially expressed genes are illustrated in Supplementary Figure S1.

## 4. Discussion

This study confirmed that (1) gene expression is heterogeneous within the same and between different AAAs and (2) global analysis of predefined gene groups should be used to investigate gene expression signaling pathways in AAA development. Enrichment of pathways containing activated genes involved in inflammation and ECM degradation, such as the chemokine and cytokine signaling pathway, as well as the integrin signaling pathway, were detected. Both have previously been reported to be involved in the regulation of inflammation [16] as well as ECM cell interaction in the context of mechanotransduction in AAA [17].

Our previous study demonstrated a significantly increased expression of genes involved in ECM degradation in AAA regions with the highest wall stress. In addition, the lowest wall stress regions were associated with a significant enrichment of inflammation regulating genes from the cytokine–cytokine receptor interaction and chemokine signaling pathway [10].

These findings could not be reproduced by our single gene expression analysis, however, global pathway analysis revealed a general upregulation of genes involved in ECM interaction (integrin signaling pathway) and inflammation regulation (Inflammation mediated by chemokine and cytokine signaling pathway).

Single gene expression profiling detected significantly increased expression levels of genes involved in inflammation and degradation of ECM components. However, these did not show a consistent or repetitive pattern of expression. Detailed analysis revealed strong inter- and intraindividual heterogeneity of gene expression patterns in AAA. This could indicate different or synchronous pathomechanisms for the development and progression of AAA. These results suggest that even within the same AAA vessel wall, different gene expression patterns are activated and coexistent.

Based on epidemiologic studies showing a significantly higher risk of AAA rupture in women, differences in disease development and progression are thought to depend on sex, hormonal status, as well as patient-specific ECM integrity, and inflammatory response [18]. Since pathophysiological development of infrarenal AAAs in inflammatory conditions and hereditary connective tissue disorders are distinct from atherosclerotic and degenerative AAAs these entities were excluded.

Comparability of gene expression data depends on a detailed description of the study group. Age (47–80 years), AAA diameter (50.3–96.6 mm), and sex (3:9 ratio) were heterogeneous in our patients, and surgical repair, i.e., AAA wall sample collection, might have occurred at different stages of AAA disease progression, which is a limitation of the study. Due to the limited sample size, we did not see gender-associated differences in gene expression patterns. It is plausible that distinct pathomechanisms involving inflammatory processes, activation of MMPs, and degradation of the ECM are time-dependent during AAA progression.

Consistent with an enzyme-linked immunoassay study quantifying IL-6, Il-1beta, and TNF-alpha in ruptured and non-ruptured AAA regions, inflammatory mediators have already been shown to be heterogeneous within the same AAA [19]. In addition, Hurks et al., found higher levels of cytokines, i.a., IL-8, inflammatory cells, micro-vessels, and active proteases such as MMP-9 in lateral AAA sites compared to ventral and dorsal segments of the same AAA [20]. While the role of inflammation in the development of AAA is generally accepted, there is currently no scientific consensus on the chronological sequence of these processes.

Some AAA candidate genes were detected among the transcripts that showed an expression difference of >20% or absolute expression differences of >50% between high- and low wall stress regions of the same AAA. In particular, MMP-7 [21], MMP-9 [22], and MMP-12 [23] are estimated to be involved in AAA progression. In our study, gene expression of these candidate genes exhibited an absolute difference in expression of more than 120% within the same AAA. Both between the lowest and highest wall stress AAA, (Table 2 and Table 3) and within a specimen of the same AAA (Table 4), we found differential expressions in genes that have been described in the context of aortic aneurysms.

Due to their key role in inflammation and ECM degradation, MMPs have frequently been considered as promising candidates for targeted therapies. Elevated levels of circulating MMP-9 have been previously reported in patients with AAA [24]. Other potential targets are agonists for PPARG, a nuclear receptor that has been implicated in AAA attenuation as well as cytokine production and inflammation regulation [25]. We demonstrated increased expression of PPARG in both the single-gene expression profiling and the significantly enriched CCKR signaling pathway.

Lillvis et al. demonstrated diverging protein levels of the *HOX* gene family in thoracic and abdominal aortic samples. Especially *HOXA4* transcription levels were decreased in AAA samples as compared to healthy abdominal aortic samples [26]. We did not observe differences in gene expressions for *HOX4A* in high or low wall stress samples (FC 0.974; *p* = 0.716) and *HOX4A* was not a component of the enriched pathway analysis.

A limitation of this study is the absence of control healthy aortic samples for a comparable RNA expression analysis, as these samples are rarely obtainable from the clinical position. Open surgical procedures of the abdominal aorta either include aneurysmatic or occlusive disease but not healthy aortas.

We did not detect specific and reproducible differences in expression patterns of AAA candidate genes with respect to regions of high wall stress determined by FEA. However, a considerably high number of genes that are associated with aortic aneurysms (see Table 2) were upregulated in high wall stress AAA regions. As a more patient-specific diagnostic tool for AAA rupture estimation, FEA model validation is ongoing. High rupture risk regions estimated by FEA contained increased histopathological degeneration compared to low rupture risk samples of the same AAA [11].

Although specific gene expression differences could not be directly correlated to AAA wall stress properties, histopathological degeneration might result from the aforementioned activation of the integrin signaling pathway and Inflammation mediated by the chemokine and cytokine signaling pathway.

Due to the large heterogeneity of the observed expression patterns, we consider pathway analyses more appropriate than single gene expression profiling analyses for AAA explorative studies. Cooperation of vascular biobanks might increase the sample size and comparability of AAA cohorts for further investigations. With larger numbers of cases, gene expression analyses should also be performed with respect to distinct aneurysm morphologies and subtypes.

## 5. Conclusions

Gene expression profiling in AAA demonstrates a strong heterogeneity of underlying changes in individual gene expression. We encourage to perform genome-wide expression profiling studies and particularly global analysis of predefined gene groups to investigate AAA disease development, progression, and potential target therapies.

## Figures and Tables

**Table 1:** Patient characteristics and parameters from FEA.

Patient Characteristics	
Age (years)	67.8 ± 10.2
Female	3
Male	9
Arterial hypertension	11
Smoking history	6
Coronary artery disease	3
Peripheral arterial disease	2
Diabetes mellitus	0
**Parameters from FEA**	
Maximal AAA diameter (mm)	65.9 ± 17.7
Intraluminal thrombus volume (cm^3^)	95.7 ± 115.7
Peak wall stress (kPa)	249.9 ± 83.2

Continuous data are presented as the means ± standard deviation; categorical data are given as the counts (n = 12), mm = millimeter, kPa = kilo Pascal.

**Table 2:** Genes upregulated in the highest wall stress AAA regions.

Gene	Name	Function	Mean Expression Value		*p*-Value	FC
			Highest Wall Stress	Lowest Wall Stress		
CD36 *	CD36 Molecule (Thrombospondin Receptor)	Receptor for various ligands, angiogenesis, inflammatory response, fatty acid metabolism	10.180	9.497	0.001	1.606
			9.340	8.675	0.004	1.586
*SCD*	Stearoyl-CoA Desaturase	Lipid biosynthesis	10.718	10.109	0.038	1.525
TREM1 *	Triggering Receptor Expressed on Monocytes 1	Stimulates neutrophil and monocyte inflammatory response, release of proinflammatory cytokines	7.865	7.340	0.029	1.439
PPARG *	Peroxisome Proliferator-Activated Receptor Gamma	Binds peroxisome proliferators and controls peroxisomal beta-oxidation of fatty acids	8.669	8.197	0.028	1.386
C5AR1 *	Complement C5a Receptor 1	Receptor for complement factor C5A, stimulating chemotaxis	9.817	9.362	0.022	1.371
ALDH1A2 *	Aldehyde Dehydrogenase 1 Family Member A2	Catalyzes the synthesis of retinoic acid from retinal	7.948	7.495	0.010	1.369
*CFD*	Complement Factor D	Catalyzes the cleavage of factor B, complement activation	12.651	12.202	0.013	1.365
*TNFAIP6*	TNF Alpha Induced Protein 6	ECM stability, inflammation	9.132	8.686	0.006	1.362
OLR1 *	Oxidized Low-Density Lipoprotein Receptor 1	Marker of atherosclerosis, inducing vascular endothelial cell dysfunction, proinflammatory responses	10.310	9.871	0.033	1.356
*TFRC*	Transferrin Receptor	Cellular iron uptake	11.144	10.711	0.025	1.350
FCGR3B *	Fc Fragment of IgG Receptor IIIb	Receptor for gamma immunoglobulins (IgG)	7.900	7.468	0.016	1.348
ABCA1 *	ATP Binding Cassette Subfamily A Member 1	Cholesteral efflux pump in the cellular lipid removal pathway	10.868	10.437	0.026	1.348
NR4A2 *	Nuclear Receptor Subfamily 4 Group A Member 2	Member of the steroid–thyroid hormone–retinoid receptor family	9.623	9.216	0.047	1.326
CEBPA *	CCAAT Enhancer Binding Protein Alpha	Cell cycle regulation, body weight homeostasis	8.909	8.510	0.042	1.319
CCL20 *	C-C Motif Chemokine Ligand 20	Immunoregulation, inflammatory processes, chemotactic activity for lymphocytes	7.841	7.441	0.042	1.319
ACSL1 *	Acyl-CoA Synthetase Long Chain Family Member 1	Lipid biosynthesis, fatty acid degradation	9.595	9.199	0.014	1.316
*COLEC12*	Collectin Subfamily Member 12	Host defense carried out by vascular endothelial cells	10.513	10.132	0.026	1.302
RNASE1 *	Ribonuclease A Family Member 1, Pancreatic	Member of the pancreatic-type of secretory ribonucleases	11.631	11.251	0.026	1.301
			10.512	10.147	0.028	1.288
*SCARB1*	Scavenger Receptor Class B Member 1	Plasma membrane receptor for high density lipoprotein cholesterol (HDL)	8.103	7.727	0.034	1.298
ALDH1A1 *	Aldehyde Dehydrogenase 1 Family Member A1	Enzyme in the pathway of alcohol metabolism	10.382	10.007	0.014	1.297
			9.930	9.555	0.039	1.296
NAMPT *	Nicotinamide Phosphoribosyl-transferase	Cytokine with immunomodulating properties	8.724	8.361	0.007	1.287
STX11 *	Syntaxin 11	Intracellular protein transport	8.681	8.319	0.013	1.285
*CRABP2*	Cellular Retinoic Acid Binding Protein 2	Associated with increased circulating low-density lipoprotein cholesterol (LDL)	8.691	8.330	0.021	1.284
*C17ORF58*	Chromosome 17 Open Reading Frame 58	Associated with posterior myocardial infarction	9.325	8.965	0.024	1.283
			8.270	7.918	0.025	1.277
			8.142	7.815	0.022	1.255
*SDCBP*	Syndecan Binding Protein	transmembrane protein traffic, neuro-, and immunomodulation	10.291	9.933	0.026	1.282
MT1G *	Metallothionein 1G	Copper homeostasis	9.268	8.920	0.047	1.273
SRGN *	Serglycin	Processing of MMP2	12.621	12.277	0.032	1.269
*LOC387934*	-	unknown	7.964	7.621	0.041	1.269
*SLC31A2*	Solute Carrier Family 31 Member 2	Copper homeostasis	8.253	7.911	0.033	1.267
KLF4 *	Kruppel Like Factor 4	Differentiation of epithelial cells	10.665	10.331	0.036	1.261
*LILRA2*	Leukocyte Immuno-globulin Like Receptor A2	Immunoreceptor expressed predominantly on monocytes and B cells	8.103	7.775	0.038	1.255
THBD *	Thrombomodulin	Binds thrombin, activation of protein C	8.801	8.477	0.021	1.251
*TSC22D2*	TSC22 Domain Family Member 2	DNA-binding transcription factor activity	8.099	7.780	0.047	1.248
			9.920	9.610	0.050	1.240
CH25H *	Cholesterol 25-Hydroxylase	Cholesterol and lipid metabolism	8.687	8.375	0.023	1.241
*SAT1*	Spermidine/ Spermine N1-Acetyltransferase 1	Regulation of the intracellular concentration of polyamines	12.621	12.313	0.003	1.239
*MXD1*	MAX Dimerization Protein 1	Mediates cellular proliferation, differentiation and apoptosis	9.092	8.784	0.022	1.238
*IVNS1ABP*	Influenza Virus NS1A Binding Protein	Various cell functions, i.a. pre-mRNA splicing	8.923	8.626	0.009	1.229
			8.271	8.006	0.008	1.202
RGS2 *	Regulator of G Protein Signaling 2	Regulation of blood pressure	12.318	12.023	0.033	1.227
*GNA13*	G Protein Subunit Alpha 13	Modulator/transducer in various transmembrane signaling systems	8.959	8.665	0.002	1.226
			9.692	9.398	0.022	1.226
*ADRB2*	Adrenoceptor Beta 2	Associated with cardiovascular disease	8.128	7.836	0.034	1.225
*ATP8B4*	ATPase Phospholipid Transporting 8B4 (Putative)	Involved in cell membrane phospholipid transport	9.236	8.945	0.003	1.224
*ZNF331*	Zinc Finger Protein 331	Transcriptional repression	8.588	8.300	0.023	1.221
*EYA2*	EYA Transcriptional Coactivator And Phosphatase 2	Eye development	8.458	8.172	0.036	1.219
*HBA1*	Hemoglobin Subunit Alpha 1	Part of Hemoglobin A	13.576	13.298	0,007	1.213
*ANKRD29*	Ankyrin Repeat Domain 29	Associated with papilloma	8.001	7.726	0.029	1.210
*CREB5*	CAMP Responsive Element Binding Protein 5	CRE-dependent trans-activator	8.297	8.024	0.005	1.208
*GALC*	Galactosylceramidase	Lysosomal catabolism of glycolipids	9.205	8.935	0.047	1.206
*TLR5*	Toll-Like Receptor 5	Activation of innate immunity and inflammatory response	7.830	7.567	0.027	1.200

Upregulated genes were determined using a paired *t*-test (significant *p*-value < 0.05), comparison of mean expression values by FC > 1.2. Genes sorted by FC in descending order. Name and function according to GeneCards (http://www.genecards.org) [15]. Mean expression values are significance analysis of microarrays (SAM)-normalized and log2 logarithmized. All significantly upregulated transcripts of a gene are displayed. FC = fold change. * marked genes have already been described in the context of aortic aneurysms (Pubmed, retrieved May 2022).

**Table 3:** Genes upregulated in the biopsies with lowest AAA wall stress regions.

Gene	Name	Function	Mean Expression Value		*p*-Value	FC
			Highest Wall Stress	Lowest Wall Stress		
SDC1 *	Syndecan 1	Cell-matrix interactions for ECM proteins	7.915	8.347	0.037	0.741
*LOC100134331*	-	unknown	6.818	7.239	0.028	0.747
*REEP1*	Receptor Accessory Protein 1	Cell surface expression of odorant receptors	7.510	7.925	0.046	0.750
*ADARB1*	Adenosine Deaminase, RNA Specific B1	pre-mRNA editing of glutamate receptor subunit B	10.794	11.196	0.023	0.757
*LBH*	Limb Bud and Heart Development	Transcriptional activator	9.722	10.090	0.016	0.775
*PTP4A3*	Protein Tyrosine Phosphatase Type IVA, Member 3	Cell signaling molecule of various cellular processes	7.549	7.909	0.009	0.779
*ITM2C*	Integral Membrane Protein 2C	Negative regulator of amyloid-beta peptide production	9.592	9.934	0.050	0.789
			9.506	9.841	0.045	0.793

Upregulated genes were determined using a paired t-test (significant *p*-value < 0.05). Comparison of mean expression values by FC < 0.8. Genes sorted by FC in ascending order. Name and function according to GeneCards (http://www.genecards.org) [15]. Mean expression values are significance analysis of microarrays (SAM)-normalized and log2 logarithmized. All significantly upregulated transcripts of a gene are displayed. FC = fold change. * marked genes have already been described in the context of aortic aneurysms (Pubmed, retrieved May 2022).

**Table 4:** Top 20 genes with the greatest expression difference between the two biopsies of each patient.

Gene	Name	Mean Expression Difference (in %)	Median (in %)
*CHGB*	Secretogranin 1	313.72	69.55
SLN *	Sarcolipin	305.47	69.44
*LOC652493*	-	220.98	60.49
*LOC652694*	-	173.06	78.26
*LOC647450*	-	168.94	62.42
NTS *	Neurotensin/Neuromedin N	163.73	42.05
*NPY*	Pro-Neuropeptide Y	162.57	47.28
MMP7 *	Matrix Metalloproteinase 7	161.54	71.57
		130.93	70.09
*LOC642113*	-	147.27	73.11
*CHGA*	Chromogranin A	144.94	23.81
*DES*	Desmin	135.70	57.83
*LOC647506*	-	134.28	57.92
MMP12 *	Matrix Metalloproteinase 12	131.27	78.06
DBH *	Dopamine Beta-Hydroxylase	130.97	33.93
APOC1 *	Apolipoprotein C1	130.10	97.25
*HS3ST2*	Heparan Sulfate Glucosamine3-O-Sulfotransferase 2	126.02	54.12
CIDEC *	Cell Death Activator CIDE-3	124.29	56.24
MMP9 *	Matrix Metalloproteinase 9	124.26	57.34
*KIAA1199*	Cell Migration InducingHyaluronidase 1	120.42	76.05

The mean expression difference was determined by the percent absolute values of the expression differences between the two biopsies of each patient. All transcripts of a gene corresponding to this are displayed. Name according to GeneCards (http://www.genecards.org) [15]. * marked genes have already been described in the context of aortic aneurysms (Pubmed, retrieved May 2022).

**Table 5:** Pathways with significant gene enrichment in GO enrichment analysis.

PANTHER Pathway	N	n	*p*	FC
Integrin signaling pathway	199	150	0.001	1.63
CCKR signaling pathway	172	131	0.011	1.60
Inflammation mediated by chemokine and cytokine signaling pathway	255	176	0.028	1.45

N = number of all genes contained in pathway; n = number of genes contained in pathway with mean expression value detected on the microarray above the background level; CCKR = cholecystokinin receptor; FC = fold change. *p*-values after Bonferroni correction for multiple testing. *p*-values <0.005 were considered statistically significant.

## Data Availability

The authors confirm that the data of the manuscript are available from the corresponding author upon reasonable request.

## Supplementary Materials

The following supporting information can be downloaded at: https://www.mdpi.com/article/10.3390/jcm11123260/s1, Figure S1. Gene Ontology (GO) enrichment analysis of all the differentially expressed genes. Vertical axis displays the percentage of significant genes corresponding to each functional type. Horizontal axis displays the GO annotation corresponding to biological process.

## References

[1] Kent K.C., Zwolak R.M., Egorova N.N., Riles T.S., Manganaro A., Moskowitz A.J., Gelijns A.C., Greco G. (2010). Analysis of risk factors for abdominal aortic aneurysm in a cohort of more than 3 million individuals. J. Vasc. Surg..

[2] Lancaster E.M., Gologorsky R., Hull M.M., Okuhn S., Solomon M.D., Avins A.L., Adams J.L., Chang R.W. (2022). The natural history of large abdominal aortic aneurysms in patients without timely repair. J Vasc. Surg..

[3] Kemmerling E.M.C., Peattie R.A. (2018). Abdominal Aortic Aneurysm Pathomechanics: Current Understanding and Future Directions. Adv. Exp. Med. Biol..

[4] Darling R.C., Messina C.R., Brewster D.C., Ottinger L.W. (1977). Autopsy study of unoperated abdominal aortic aneurysms. The case for early resection. Circulation.

[5] Hellenthal F.A., Buurman W.A., Wodzig W.K.W.H., Schurink G.W.H. (2009). Biomarkers of AAA progression. Part 1: Extracellular matrix degeneration. Nat. Rev. Cardiol..

[6] Dihlmann S., Erhart P., Mehrabi A., Nickkholgh A., Lasitschka F., Böckler D., Hakimi M. (2014). Increased Expression and activation of Absent in Melanoma 2 Inflammasome components in lymphocytic infiltrates of abdominal aortic aneurysms. Mol. Med..

[7] Michel J.B., Martin-Ventura J.L., Egido J., Sakalihasan N., Treska V., Lindholt J., Allaire E., Thorsteinsdottir U., Cockerill G., Swedenborg J. (2011). Novel aspects of the pathogenesis of aneurysms of the abdominal aorta in humans. Cardiovasc. Res..

[8] Reeps C., Pelisek J., Seidl S., Schuster T., Zimmermann A., Kuehnl A., Eckstein H.H. (2009). Inflammatory infiltrates and neovessels are relevant sources of MMPs in abdominal aortic aneurysm wall. Pathobiology.

[9] Humphrey J.D., Schwartz M.A., Tellides G., Milewicz D.M. (2015). Role of mechanotransduction in vascular biology: Focus on thoracic aortic aneurysms and dissections. Circ. Res..

[10] Erhart P., Schiele S., Ginsbach P., Grond-Ginsbach C., Hakimi M., Böckler D., Lorenzo-Bermejo J., Dihlmann S. (2017). Gene Expression Profiling in Abdominal Aortic Aneurysms After Finite Element Rupture Risk Assessment. J. Endovasc. Ther..

[11] Erhart P., Grond-Ginsbach C., Hakimi M., Lasitschka F., Dihlmann S., Böckler D., Hyhlik-Dürr A. (2014). Finite element analysis of abdominal aortic aneurysms: Predicted rupture risk correleates with aortic wall histology in individual patients. J. Endovasc. Ther..

[12] Ashburner M., Ball C.A., Blake J.A., Botstein D., Butler H., Cherry J.M., Davis A.P., Dolinski K., Dwight S.S., Eppig J.T. (2000). Gene ontology: Tool for the unification of biology. The Gene Ontology Consortium. Nat. Genet..

[13] Gene Ontology Consortium (2021). The Gene Ontology resource: Enriching a GOld mine. Nucleic Acids Res..

[14] Mi H., Ebert D., Muruganujan A., Mills C., Albou L.-P., Mushayamaha T., Thomas P.D. (2021). PANTHER version 16: A revised family classification, tree-based classification tool, enhancer regions and extensive API. Nucleic Acids Res..

[15] Safran M., Rosen N., Twik M., BarShir R., Stein T.I., Dahary D., Fishilevich S., Lancet D. (2021). The GeneCards Suite. Practical Guide to Life Science Databases.

[16] Prucha M., Sedivy P., Stadler P., Zdrahal P., Matoska V., Strnad H. (2019). Gene expression in patients with abdominal aortic aneurysm-more than immunological mechanisms involved. Physiol Res..

[17] Sun Z., Guo S.S., Fässler R. (2016). Integrin-mediated mechanotransduction. J. Cell Biol..

[18] Villard C., Roy J., Bogdanovic M., Eriksson P., Hultgren R. (2021). Sex hormones in men with abdominal aortic aneurysm. J. Vasc. Surg..

[19] Wilson W.R.W., Wills J., Furness P.N., Loftus P.N., Thompson M.M. (2010). Abdominal aortic aneurysm rupture is not associated with an Up-regulation of inflammation within the aneurysm wall. Eur. J. Vasc. Endovasc. Surg..

[20] Hurks R., Pasterkamp G., Vink A., Hoefer I.E., Bots M.L., van de Pavoordt H.D., de Vries J.P., Moll F.L. (2012). Circumferential heterogeneity in the abdominal aortic aneurysm wall composition suggests lateral sides to be more rupture prone. J. Vasc. Surg..

[21] Zhang W.-H., Qiao C.H., Zhang X., Luo H., Sun X.-K. (2017). The expression of MMP-7 in serum and aneurysm tissues of patients with abdominal aortic aneurysm associated with hypertension and the clinical efficacy of endovascular exclusion. Eur. Rev. Med. Pharmacol. Sci..

[22] Elmore J.R., Keister B.F., Franklin D.P., Youkey J.R., Carey D.J. (1998). Expression of matrix metalloproteinases and TIMPs in human abdominal aortic aneurysms. Ann. Vasc. Surg..

[23] Longo G.M., Buda S.J., Fiotta N., Xiong W., Griener T., Shapiro S., Baxter B.T. (2005). MMP-12 has a role in abdominal aortic aneurysms in mice. Surgery.

[24] Li T., Jiang B., Li X., Sun H.-Y., Li X.-T.L., Jing J.-J., Yang J. (2018). Serum matrix metalloproteinase-9 is a valuable biomarker for identification of abdominal and thoracic aortic aneurysm: A case-control study. BMC Cardiovasc. Disord..

[25] Hwang J.S., Kim H.J., Kim G., Kang E.S., Ham S.A., Yoo T., Paek K.S., Yabe-Nishimura C., Kim H.J., Seo H.G. (2014). PPARδ reduces abdominal aortic aneurysm formation in angiotensin II-infused apolipoprotein E-deficient mice by regulating extracellular matrix homeostasis and inflammatory responses. Int. J. Cardiol..

[26] Lillvis J.H., Erdman R., Schworer C.M., Golden A., Derr K., Gatalica Z., Cox L.A., Shen J., Vander Heide R.S., Lenk G.M. (2011). Regional expression of HOX4A along the aorta and ist potential role in human abdominal aortic aneurysms. BMC Physiol..

