# Supplementary material for: Gene Expression Profiling in Abdominal Aortic Aneurysms
Source: J Clin Med. 2022 Jun 7;11(12):3260. doi: 10.3390/jcm11123260 (PMC9225238; doi:10.3390/jcm11123260)
Supplement: Supplementary file 1 [file jcm-11-03260-s001.zip › jcm-1658895-supplementary.pdf]

Supplementary Figure S1. Gene Ontology (GO) enrichment analysis of all the differentially expressed genes. Vertical axis displays the percentage of significant genes corresponding to each functional type. Horizontal axis displays the GO annotation corresponding to biological process.

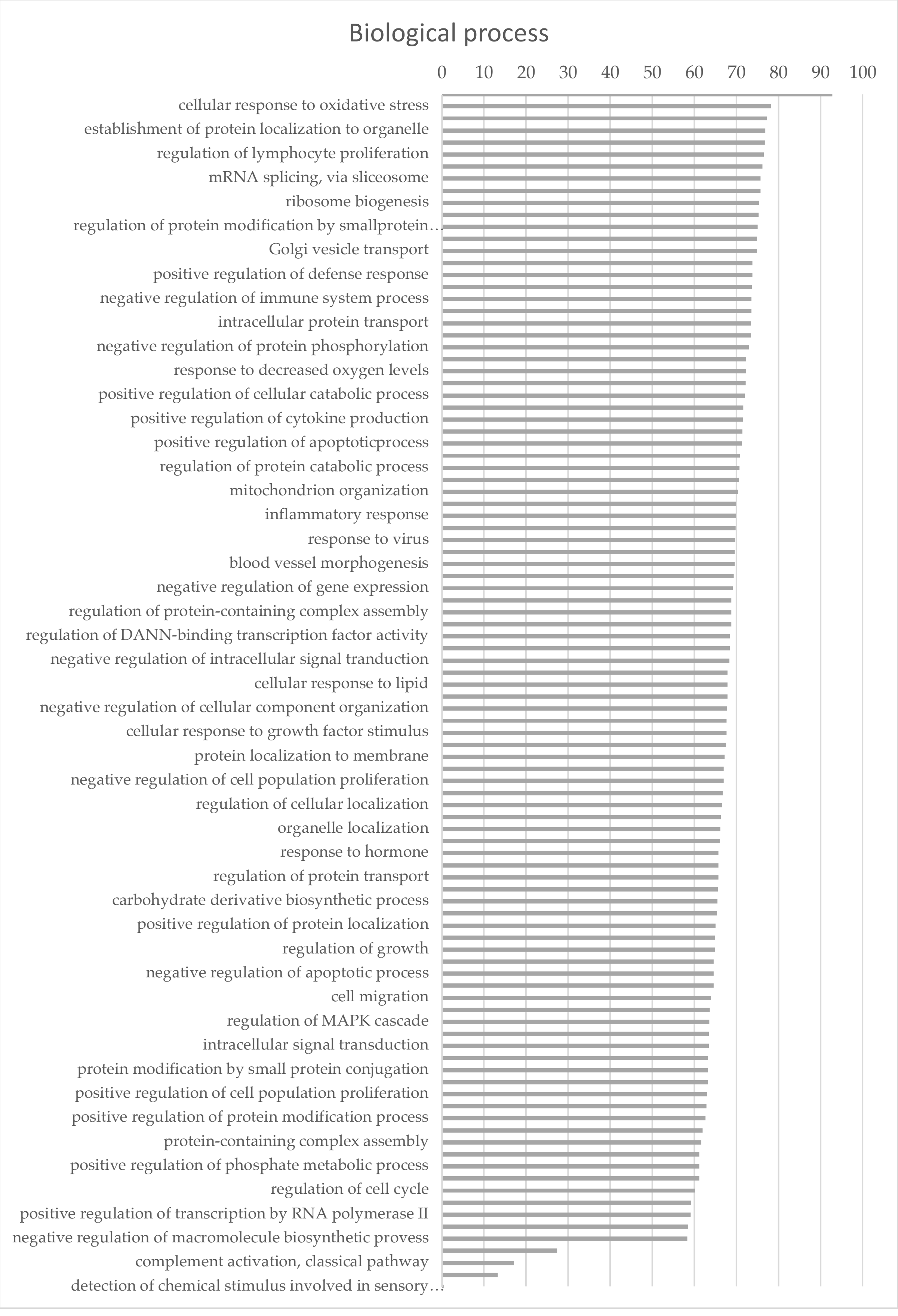

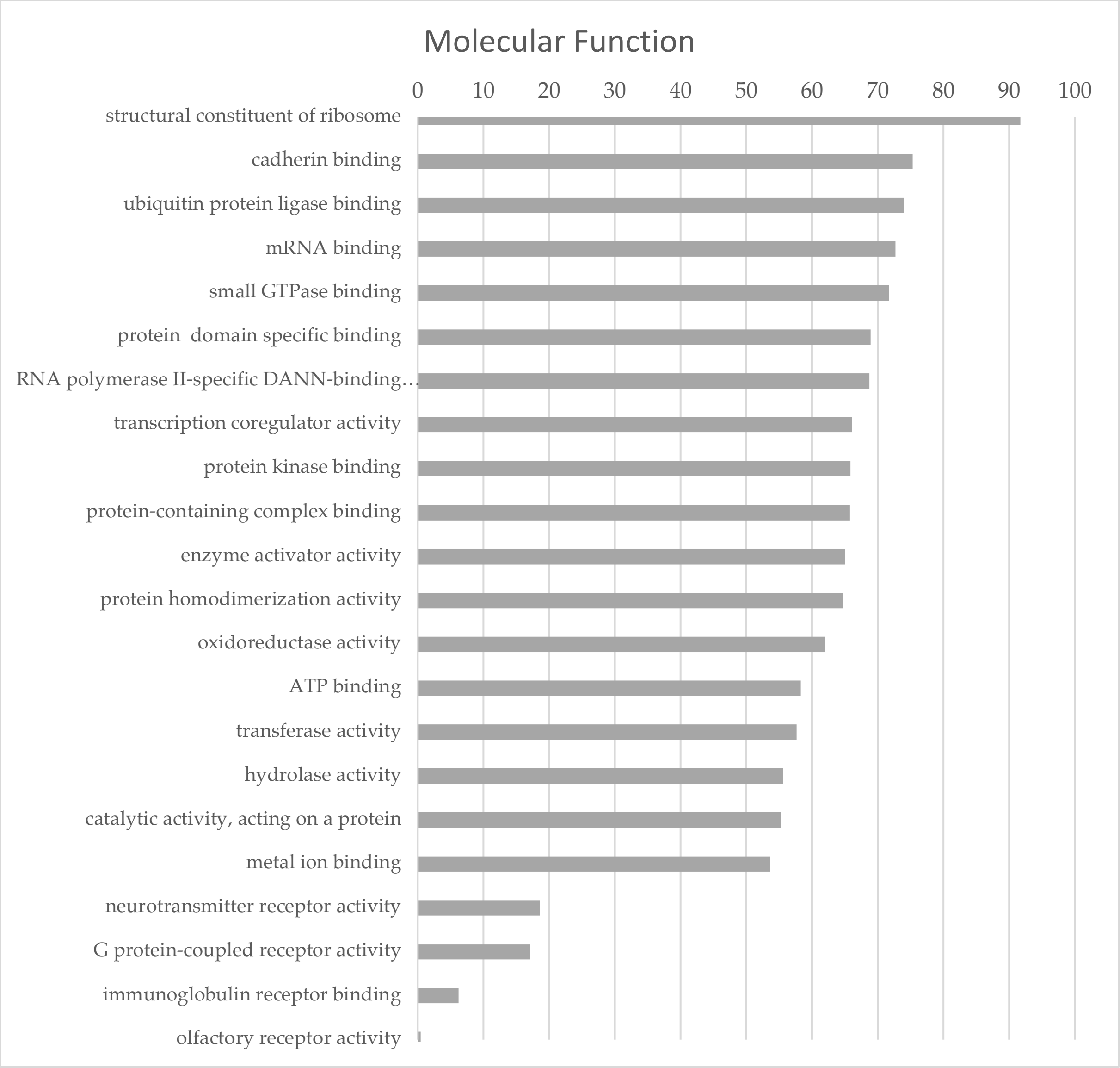
Molecular Function
0
10
20
30
40
50
60
70
80
90
100
structural constituent of ribosome
cadherin binding
ubiquitin protein ligase binding
mRNA binding
small GTPase binding
protein domain specific binding
RNA polymerase II-specific DANN-binding…
transcription coregulator activity
protein kinase binding
protein-containing complex binding
enzyme activator activity
protein homodimerization activity
oxidoreductase activity
ATP binding
transferase activity
hydrolase activity
catalytic activity, acting on a protein
metal ion binding
neurotransmitter receptor activity
G protein-coupled receptor activity
immunoglobulin receptor binding
olfactory receptor activity

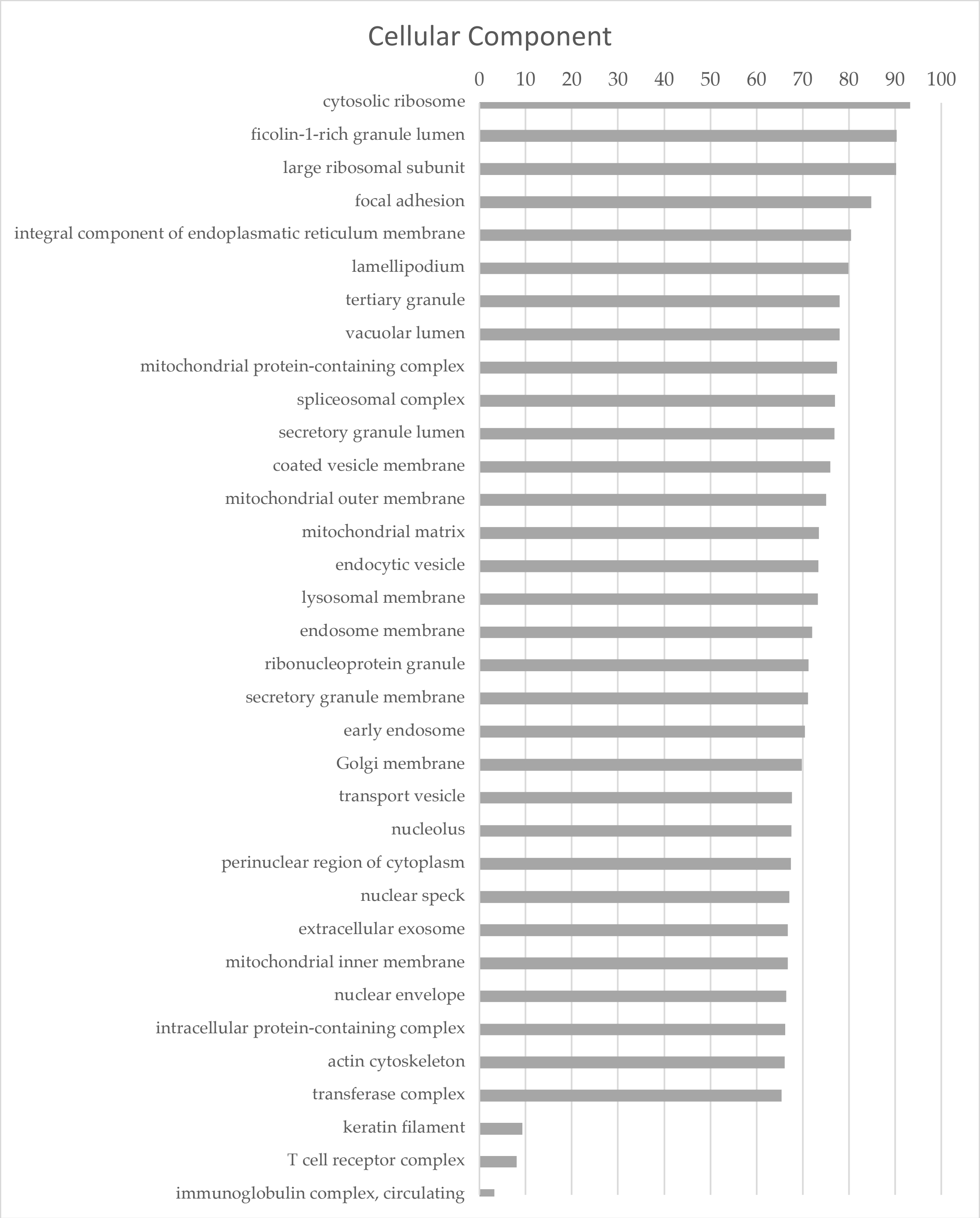
Cellular Component
0
10
20
30
40
50
60
70
80
90
100
cytosolic ribosome
ficolin-1-rich granule lumen
large ribosomal subunit
focal adhesion
integral component of endoplasmatic reticulum membrane
lamellipodium
tertiary granule
vacuolar lumen
mitochondrial protein-containing complex
spliceosomal complex
secretory granule lumen
coated vesicle membrane
mitochondrial outer membrane
mitochondrial matrix
endocytic vesicle
lysosomal membrane
endosome membrane
ribonucleoprotein granule
secretory granule membrane
early endosome
Golgi membrane
transport vesicle
nucleolus
perinuclear region of cytoplasm
nuclear speck
extracellular exosome
mitochondrial inner membrane
nuclear envelope
intracellular protein-containing complex
actin cytoskeleton
transferase complex
keratin filament
T cell receptor complex
immunoglobulin complex, circulating
